# Larch Bark in Hæmoptysis

**Published:** 1860-09

**Authors:** 


### Larch Bark in Hæmoptysis.

The use of tincture of larch bark in pulmonary hemorrhage is mentioned in the *American Druggists' Circular*, for August, with a short report of several cases successfully treated with this remedy, from the *Med. Times & Gazette*, by Dr. Owen Daly. It was used in the dose of twenty drops every third hour.

